# Blood Flow Restricted Exercise and Vascular Function

**DOI:** 10.1155/2012/543218

**Published:** 2012-10-22

**Authors:** Masahiro Horiuchi, Koichi Okita

**Affiliations:** ^1^Department of Physiology, Yamanashi Institute of Environmental Sciences, Kami-yoshida 5597, Fuji-yoshida, Yamanashi 4030005, Japan; ^2^Northern Regions, Life long Sports Research Center, Hokusho University, Bunkyoudai 23, Ebetsu, Hokkaido 0698511, Japan; ^3^Department of Sports Education, Hokusho University, Bunkyodai 23, Ebetsu, Hokkaido 0698511, Japan

## Abstract

It is established that regular aerobic training improves vascular function, for example, endothelium-dependent vasodilatation and arterial stiffness or compliance and thereby constitutes a preventative measure against cardiovascular disease. In contrast, high-intensity resistance training impairs vascular function, while the influence of moderate-intensity resistance training on vascular function is still controversial. However, aerobic training is insufficient to inhibit loss in muscular strength with advancing age; thus, resistance training is recommended to prevent sarcopenia. Recently, several lines of study have provided compelling data showing that exercise and training with blood flow restriction (BFR) leads to muscle hypertrophy and strength increase. As such, BFR training might be a novel means of overcoming the contradiction between aerobic and high-intensity resistance training. Although it is not enough evidence to obtain consensus about impact of BFR training on vascular function, available evidences suggested that BFR training did not change coagulation factors and arterial compliance though with inconsistence results in endothelial function. This paper is a review of the literature on the impact of BFR exercise and training on vascular function, such as endothelial function, arterial compliance, or other potential factors in comparison with those of aerobic and resistance training.

## 1. Introduction

“A man is as old as his arteries” was a favorite axiom of William Osler (1849–1919), sometimes called the “Father of Modern Medicine,” and to some extent accurately represents the effect of vascular dysfunction on various aging processes [1]. To date, it has been recognized that arterial dysfunction, such as increased arterial stiffness, is closely associated with the pathogenesis of cardiovascular disease, which in turn increases mortality by increasing the risk of events such as myocardial infarction and stroke [2–4]. A higher physical activity level as well as regular exercise may be effective at diminishing the risk of coronary heart disease [5, 6] and stroke [7, 8].

From the standpoint of exercise physiology, exercise is categorized as aerobic and resistance exercise. Briefly, aerobic exercise is a physical exercise of relatively low intensity that depends primarily on the aerobic energy-generating process, for example, running and leg cycling [9]. In contrast, resistance exercise is also physical exercise of relatively moderate and higher intensity that uses a resistance to the force of muscular contraction, in other words, strength training [10].

Although aerobic exercise may improve arterial function [11], it has also been reported that aerobic exercise is insufficient to inhibit the loss in muscular strength that comes with advancing age [12, 13]. Resistance exercise is recommended to prevent sarcopenia, age-induced muscular degeneration which often entails reduced activities of daily living (ADLs) [14]. According to the guideline of American College of Sports Medicine (ACSM), a mechanical load greater than 70% of the one-repetition maximum load (1 RM) can produce morphological and functional muscular adaptations [14]. However, these higher-load exercises are frequently associated with orthopedic complications [15, 16]. In addition, it has been reported that high-intensity resistance training (>80% of 1 RM) reduces central artery compliance [15]. These findings suggest that such a high-intensity resistance exercise should be prescribed carefully, particularly for aged people and patients with cardiovascular disease. Recently, several studies have demonstrated that low-intensity resistance exercise with blood flow restriction (BFR) [17–23] and BFR walking [24] dramatically leads to muscle hypertrophy and strength gain and that it results in adaptations equal to those of high-intensity resistance training [22]. Although the effect of resistance exercise with BFR and BFR walking on vascular function is still unclear, there is a possibility that this exercise modality can be an important therapeutic prescription not only for sarcopenia but also for vascular dysfunction because of the lower exercise intensity compared to high-intensity resistance training. In this review, we would like to focus on the impact of such exercise on vascular function in comparison with the effects of aerobic and resistance training alone and in combination.

## 2. Evaluation for Vascular Function

In human studies, as it is almost impossible to evaluate large arterial function directly, various noninvasive methodologies have been used to evaluate arterial function in humans. In this section, we introduced several methodologies, which have investigated the impact of BFR exercise and training.

### 2.1. Arterial Compliance and Stiffness

Generally, arterial compliance can be measured by a combination of ultrasound imaging of any artery, for example, carotid artery, with simultaneous applanation of tonometrically obtained arterial pressure from the contralateral artery, permits noninvasive determination of arterial compliance[11]. This methodology can be applied to any artery, which can measure pulse wave, for example, radial and femoral arteries. In addition to arterial compliance [25], *β*-stiffness index provides an indicator of arterial compliance adjusted for distending pressure [26].

Arterial compliance decreases with advancing age [27–29], and these reductions are associated with isolated systolic hypertension, accompanied with left ventricular hypertrophy [30]. Indeed, several studies demonstrated that decreased arterial compliance and/or increased arterial stiffness have been identified as independent risk factors of cardiovascular disease [27, 31–36].

### 2.2. Ankle Brachial Index

Although ankle and brachial blood pressure indicates similar value in healthy humans, under continued occlusion and/or stenosis in lower limbs induced by arteriosclerosis, ankle blood pressure would decrease compared with brachial blood pressure. Therefore, ankle-brachial blood pressure index (ABI) is a typical indicator for screening peripheral arterial disease (PAD). Indeed, diagnostic accuracy for stenosis above 50% in leg arteries in PAD patients showed excellent values, that is, sensitivity is 90% and specificity is 98%, respectively [37–39]. Generally, ABI is thought to be a predictor for screening in PAD patients. However, ABI is simple, inexpensive, and noninvasive methodology, and it is also reported that ABI is a good predictive factor for coronary arterial disease [40], suggesting that this indicator can be useful test for arterial dysfunction.

### 2.3. Pulse Wave Velocity

Arterial stiffness is defined by a decrease in aortic distensibility. In human clinical studies, the measurement of the pulse wave velocity (PWV) has been broadly used and generally accepted as the gold standard to evaluate aortic distensibility [41]. PWV is calculated by dividing the distance between any two different arteries, for example, carotid and femoral arteries, by the traveled time in the pulse wave from one site to the other site in arteries [42, 43]. PWV is inversely related to caliber of a blood vessel and blood viscosity and is proportional to vessel wall thickness and distensibility, indicating that higher speed of PWV reflects lower aortic distensibility. Clinically, the PWV is closely associated with pathogenesis in cardiovascular disease [32–36, 44–46].

### 2.4. Flow Mediated Dilation

As endothelial dysfunction precedes arteriosclerosis, assessment of endothelium-mediated vasodilator function has also been widely used to evaluate endothelial function [47, 48]. In general, flow-mediated dilation (FMD) can be described by any vasodilatation of an artery following an increase in luminal blood flow and internal-wall shear stress induced by reactive hyperemia. The principles, assessment, and evaluation are stated in some excellent reviews [49, 50], briefly, after several minutes of arterial cuff occlusion at proximal or distal portion in any artery, for example, brachial and popliteal artery, immediate cuff deflation can lead to increase shear stress induced by reactive hyperemia and activate endothelial nitric oxide (NO) synthase (eNOS). This activation leads to a shear-stress-mediated augmented NO production in endothelial cells. FMD is calculated as the difference between the maximum diameter and during reactive hyperemia and baseline diameter, and it is expressed as the relative change (%). Since previous studies have shown that brachial artery FMD is emerging as an independent predictor of future cardiac events [51–53], FMD can be a good predictor indicating that this assessment can be a good noninvasive marker of local NO bioavailability in the endothelial cell, which is an important factor and predictor in protecting against cardiovascular disease.

## 3. Impact of Aerobic Exercise and Training on Vascular Function

Aerobic capacity determined by maximal oxygen uptake (${\dot{\text{V}}\text{O}}_{2\max}$) is strongly associated with the risk of cardiovascular disease [54, 55], and an inverse relationship has been observed between ${\dot{\text{V}}\text{O}}_{2\max}$ and PWV [56]. Since aerobic capacity is improved by regular aerobic training, to date, numerous studies have demonstrated the influence of regular aerobic training on vascular function in athletes, sedentary subjects, and aged people. Decreased arterial stiffness has been observed in cyclists, middle- or long-distance runners, and triathletes compared with sedentary subjects [57]. It was also reported that collegiate middle- or long-distance runners had lower arterial stiffness [58, 59] and middle-aged and older athletes had more distensibility in their central arteries compared with age-matched sedentary people [11, 60]. Moreover, in healthy young men [61] and postmenopausal women [62], regular aerobic exercise reduced arterial stiffness.

In addition to these cross-sectional studies, intervention studies of regular aerobic training also revealed an improvement in arterial stiffness and endothelial function. Despite differences in the training period from 4 to 16 weeks, regular aerobic training improved arterial stiffness in young healthy subjects [61, 63] and in middle- and older-aged people [11]. It was also reported that 16 weeks of regular aerobic training improved PWV in middle- and older-aged people [64] and pre- and stage 1 hypertensive patients [65]. Moreover, several studies have demonstrated that regular aerobic exercise improved endothelium-dependent vasodilation. Higashi et al. [66] revealed that regular aerobic exercise reduced resting blood pressure and improved endothelium-dependent vasodilatation in essential hypertensive patients. Similar results were observed in healthy subjects [67], diabetic patients [68], and those with coronary arterial disease [69], respectively. Interestingly, daily physical activity level was also associated with ABI [70], intima-media thickness [71], and PWV [72, 73]. To the best of our knowledge, one study demonstrated that endurance athletes showed a higher PWW compared to recreational active control subjects [74]. Although this mechanism is unclear, it is assumed that repeated and excessive training stress due to lower resting heart rate, resulting in increased stroke volume, imposed on the elastic component, may cause mechanical fatigue of the arterial wall. However, this is clearly insufficient evidence to support that aerobic exercise impairs vascular function because the results emerged from extremely trained athletes and cross-sectional study. Taken together, continued aerobic exercise training may improve vascular function assessed by various methodologies even though with one exception [74].

## 4. Impact of Resistance Exercise and Training on Vascular Function

During resistance exercise, for example, weight lifting, both systolic and diastolic blood pressures increase dramatically [75, 76], whereas only systolic blood pressure increases during aerobic exercise, without an accompanying increase in diastolic blood pressure [77, 78]. Thus, in the classical paradigm, resistance training would not be recommended for aged people, and in particular, for patients with cardiovascular disease. However, recent studies have revealed that aerobic training alone cannot maintain muscle volume and strength, which are needed to prevent sarcopenia [12, 13], the degenerative loss of skeletal muscle with advanced age, often leading to a bed-ridden lifestyle with reduced activities of daily living (ADLs). Resistance exercise is widely recommended to protect against metabolic syndrome, because such exercise can increase muscle strength and volume and may have a positive effect on glucose metabolism, blood lipids, and basal metabolic rate [13, 14]. Its value for older-aged subjects at risk of sarcopenia or vascular disease thus seems potentially significant.

However, because of conventional wisdom regarding the dangers of resistance training, studies about the impact of resistance training on vascular function are ten years behind where they might be today. Bertovic et al. [79] revealed that aortic distensibility in resistance-trained men was lower than that in age-matched control subjects. It was also reported that age-related arterial stiffness is more pronounced in strength-trained men than in age-matched sedentary subjects [15]. However, results of more recent studies, which investigated effects of resistance exercise and training on vascular function, seem to be controversial. Greater arterial stiffness was also observed in strength-exercised athletes than in sedentary people [58, 59, 80]. It was also reported that greater age-related reduction in arterial compliance in resistance-trained men was observed compared to sedentary men [81]. In contrast, arterial stiffness assessed by central/peripheral PWV did not differ between highly resistance trained and sedentary men [82]. It was also reported that ischemic reperfusion injury induced by 20-min cuff occlusion significantly reduced brachial artery FMD in sedentary young men but unchanged in resistance-trained adults [83]. Phillips et al. [84] demonstrated that resistance and endurance trained subjects showed a similar responses in FMD to acute impairment of endothelial dysfunction induced by single weight lifting. Moreover, Fahs et al. [85] demonstrated that a significant inverse association between muscular strength and central PWW, independent of aerobic fitness, suggested that resistance training, which can improve muscular strength, might improve aortic stiffness [85].

In addition to these cross-sectional studies, an intervention study found that four months' resistance training reduced arterial compliance in young men [15]. In contrast, it was reported that resistance training did not decrease arterial compliance in young men [86], in premenopausal women [87], and in elderly men [88]. In addition, resistance training did not alter resting and postexercise aortic blood pressure and wave reflection in middle-aged women [89]. It has been suggested that continued resistance exercise did not affect endothelial function assessed by FMD in young men [90] and postmenopausal women [91]. However, shorter period of resistance training, that is, four weeks, improved peak forearm blood flow induced by reactive hyperemia but increased arterial stiffness in pre- and stage-1 hypertensives [65]. Rakobowchuk et al. [92] demonstrated that FMD responses were not altered with resistance training, however, brachial artery vessel diameter increases and postocclusion blood flow increases with this training modality. These results may have an important role in clinical application because resistance training can be a stimulator that may enhance resistance vessel function. Indeed, basal limb blood flow and vascular conductance were improved with resistance training [93]. Similarly, slow movement resistance training increased basal limb blood flow as well as resistance training at normal speed [94], and weight training increased forearm blood flow as well as aerobic exercise in healthy males [95]. A recent finding also indicated that resting forearm blood flow and peak blood flow in response to reactive hyperemia significantly increased by resistance training in overweight and obese women [96].

From the point view of comparison in different populations, it was reported that decreased augmentation index was observed in old women, but not in old men [97]. Heffernan et al. [98] indicated that resistance training leads to increase in microvascular endothelial function in African-American and white men, while it increased brachial stiffness in only African-American. Additionally, resistance training improved endothelium-dependent vasodilatation as well as aerobic training and aerobic plus resistance training in patients with recent myocardial infarction, indicating that these improvements were independent of exercise type [99].

Recently, the effects of combined exercise, that is, aerobic and resistance training on vascular function have been elucidated [100, 101]. Combined training consisting of high-intensity resistance training, followed by 30-min aerobic leg cycling, demonstrated a slight increase in carotid arterial compliance (*P* = 0.06) in young men [100]; furthermore, moderate-intensity combined circuit resistance exercise and endurance exercise improved arterial stiffness in postmenopausal women [101].

However, from the viewpoint of elderly health, it may not be necessary to use higher loads, for example, >80% 1 RM. Thus, recent studies have investigated the influence of moderate-intensity resistance exercise on vascular function. It was reported that three months' moderate-intensity resistance exercise training did not alter brachial-ankle (ba) PWV [102], arterial compliance [103], or arterial stiffness [104]. Moreover, 1-year resistance training intervention in overweight women significantly improved FMD [105], and low-intensity resistance training with short interset rest period improved baPWV and FMD in young subjects [106]. Similarly, moderate-intensity resistance training for short period, that is, four weeks training, improved FMD responses in end-stage heart failure patients [107].

Although the physiological mechanisms underlying the vascular function, such as arterial stiffness, compliance, and endothelial function assessed by FMD, associated with resistance training are unclear, it is well known that higher-intensity resistance training may be a potent stimulator to increase sympathetic nervous system activity [108, 109]. Augmented sympathetic nerve activity may act to increase arterial stiffness by providing chronic restraint on the arterial wall via greater sympathetic adrenergic vasoconstrictor tone [110]. During resistance exercise, for example, weight lifting, both systolic and diastolic blood pressures increase dramatically [75, 76]. These acute elevated blood pressures during resistance exercise may alter the arterial structure, and/or arterial load-bearing properties, resulting in arterial stiffness increase and/or impaired reactive hyperemic blood flow with repeated exposure. One interesting and supporting finding was that upper body resistance training group increased arterial stiffness but unchanged with lower limb resistance training group and sedentary control subjects [111]. In their study, norepinephrine concentration (NE) significantly increased in only upper body training group after 10-week training. As it is established that NE release is strongly related to the changes in absolute levels of sympathetic nerve activity [112], elevated NE concentration suggested that resting sympathetic nerve activity increased by 10-week upper body resistance training, resulted in that increased arterial stiffness caused by alteration arterial structure, and/or arterial load-bearing properties.

Given these numerous previous studies, it is still unclear and in controversy whether resistance training increases arterial stiffness and/or diminish endothelial function. It might be possible that effect of resistance exercise on vascular function may be affected by various factors, such as exercise intensity, different populations, and exercise modality.

## 5. Impact of Blood Flow Restricted Exercise and Training on Vascular Function

According to the guidelines of the ACSM, resistance training at 70% or greater of 1-RM is recommended in order to achieve muscle hypertrophy and increased strength [14]. However, it is difficult for certain individuals, such as the elderly and rehabilitating athletes, to use such a high-intensity load. In recent years, studies on one alternative, namely, low-intensity resistance training with BFR and BFR walking, have provided compelling data that such training leads to muscle hypertrophy and strength increases [17, 19–22, 24, 113–117] and results in adaptations equal to those of high-intensity resistance training [21, 22]. As the universal way of this exercise modality seems not to be established, applied cuff pressure and exercise intensity are varied. The underlying principle of this unorthodox technique is that occlusive cuff pressure is greater above individual's systolic blood pressure and exercise intensity is 20–30% of maximal voluntary contraction [114]. During BFR exercise, cuff pressure occludes venous return and causes arterial blood flow to become turbulent, resulting in the enhanced metabolic stress and fast-twitch fiber recruitment in skeletal muscle [118]. At the end of exercise, ischemic reperfusion induced by cuff deflation stimulates shear stress, followed by greater vasodilatation and/or enhanced blood flow [119].

Although the precise mechanism by which BFR exercise produces muscle hypertrophy is still unclear, it was reported that low-intensity exercise with BFR can increase that rate of muscle protein synthesis and stimulate mammalian target of rapamycin complex 1 (mTORC1) and MAPK-mediated anabolic signaling [120]. However, a recent study revealed that reactive hyperemic blood flow is not a primary mechanism for BFR exercise-induced mTORC1 signaling and muscle protein synthesis [121]. Another potential factor to induce muscle hypertrophy may be an intramuscular metabolic stress such as depletion of phosphocreatine, an increase in inorganic phosphate, and a decrease in muscle pH [118]. We recently investigated the effects of short-term resistance training with BFR on muscle mass and strength, and found that elevated metabolic stress may be a crucial factor in obtaining successful results from resistance training with BFR [122]. Taken together, it is likely that BFR training can produce muscle hypertrophy without the effect of reactive hyperemic blood flow. However, since hyperemic blood flow per se may be induced by greater shear stress and this greater shear stress induces vasodilatation, which results in increased nitric oxide [49, 50], moreover, maximal dilation was observed after ischemic exercise [123], there might be a possibility that BFR exercise and training would have a beneficial effect on vascular function such as arterial compliance and endothelial function, indeed, observed.

Tables 1 and 2 summarize studies undertaken to investigate the effects of acute or chronic BFR exercise on vascular function such as arterial compliance, endothelial function, and related biomarkers. It has been difficult to obtain consensus due to the large number of variables such as age, gender, intensity and exercise modality, intervention period, applied cuff pressure and cuff width, and evaluation of vascular function, all of which may influence the training effect. We discuss the influence of BFR exercise and training on vascular function based on current findings in the following session.

### 5.1. Impact of Blood Flow Restricted Exercise on Arterial Compliance or Stiffness

Fahs et al. [124] reported that acute knee extension and flexion with BFR increased arterial compliance without changes in vascular conductance and that this increase was similar to low-intensity knee extension without BFR. They suggested that this acute increase in arterial compliance may be attributed to augmented regional vasoactive substances [125], and decreased systemic sympathetic vasoconstrictor tone [126].

Additionally, several interventional studies have indicated the influences of BFR training on arterial compliance. Kim et al. [127] reported that arterial compliance assessed in the radial artery of young men did not change by low-intensity resistance training with BFR for 3 weeks. Similarly, four-weeks BFR training did not alter arterial stiffness evaluated by the PWV between the femoral and tibial arteries in young male subjects [128]. Fahs et al. [129] extended the training period from 3 to 6 weeks and observed no change in arterial compliance assessed by the same device that had been used in the previous study [127]. Interestingly, one recent study [130] revealed that low-intensity (30% 1 RM) upper-body BFR exercise training, that is, bench press did not diminish carotid artery compliance, while high-intensity bench press training without BFR decreased arterial compliance.

The underlying physiological mechanism(s) associated with unaltered arterial compliance, that is, four intervention studies, and with increased arterial compliance, that is, one cross-sectional study, is unclear. Impaired arterial compliance induced by high-intensity resistance training without BFR may be attributed to acute elevated blood pressure via increasing sympathetic nerve activity system [108–110], resulting in alteration arterial structure and/or arterial load-bearing properties. These changes may be related to impaired arterial compliance. In the previous studies of BFR training, resting blood pressure did not change after several weeks intervention with BFR resistance training [127, 129]. Moreover, the resistance training with and without BFR-induced carotid arterial compliance changes was associated with changes in systolic blood pressure during training intervention [130], indicating that elevation blood pressure may play a role to induce arterial compliance changes during BFR training. Conversely, postexercise hypotension did not occur after low-intensity resistance training with BFR, while high-intensity resistance training elicited greater postexercise hypotension [131]. Postexercise hypotension can be considered an important strategy to control resting blood pressure, especially in hypertensive patients [132, 133]. Therefore, it may be reasonable that BFR training did not change arterial compliance based on unobserved postexercise hypotension with acute BFR exercise [131].

In addition to these resistance exercise and training with BFR, effect of chronic BFR walking on arterial compliance has been reported. Ozaki et al. [134] revealed that carotid arterial compliance significantly improved by 10 weeks of BFR walking in elderly people. The interesting considerations regarding their study are that the training period was longer than in previous studies (10 weeks versus 3 to 6 weeks), and the participants were elderly, with the majority being women (3 men and 10 women). This result might have great clinical relevance in understanding the potential application of BFR training. Recently, age- and sex-related differences in cardiovascular responses both at rest and during exercise have been elegantly reviewed [135]. For example, (1) reducing oxidative stress improves carotid arterial compliance in postmenopausal women but not older men, (2) habitual exercise abolished age-related differences in central arterial stiffness in older women, (3) vasoconstrictor responses to sympathetic stimulation is blunted in women compared to men (see [135] in detail). These gender differences in cardiovascular responses and adaptation to exercise training might affect vascular function in response to BFR exercise and training though only one study showed that carotid arterial compliance was improved by BFR training in elderly women.

It is well known that regular aerobic training improves carotid arterial compliance [11], whereas high-intensity resistance training reduces arterial compliance [15]. Although the mechanism of improved arterial compliance in elderly women is still unclear, there are several possible physiological mechanisms to explain these changes. A previous study demonstrated that BFR training-induced improvement in carotid arterial compliance was approximately 30% [134]. In the previous cross-sectional study, carotid arterial compliance in middle-aged and older men is 20–35% higher than that in age-matched recreational and sedentary subjects [11]. They also conducted interventional study and demonstrated that 13.5 weeks of aerobic training produced a 25% increase in carotid arterial compliance. Similarly, carotid artery compliance in postmenopausal women increased about 40% following 3 months of home-based regular aerobic exercise [136]. Abe et al. [137] reported that 6 weeks of BFR walking training did not improve peak aerobic capacity, but it significantly increased muscular size and strength in elderly subjects (2 men and 9 women). Thus, it might be significant that arterial compliance was improved in elderly women, probably without systemic aerobic capacity improvement. However, only study revealed these results, hence, future studies are needed whether these improvements are specific for elderly women.

Taken together, BFR exercise and training did not change arterial compliance, while only two studies showed that acute BFR exercise and chronic BFR walking improved arterial compliance [124, 134]. However, due to insufficient evidence, at least what we can say is that BFR training including resistance exercise and walking may not worse arterial compliance, possibly, associated with unchanged resting blood pressure with BFR resistance training [127, 129] and BFR walking [134].

### 5.2. Influence of BFR Training and Walking on Endothelial Function

Endothelium-dependent vasodilatation in conduit artery, for example, brachial and popliteal arteries, can be evaluated by FMD, which is a good in protecting against cardiovascular disease [51–53].

BFR exercise can lead to reactive hyperemic blood flow [119] and increased microvascular filtration capacity induced by ischemic reperfusion [138]. Thus, it might be possible that these physiological responses may lead to an enhanced vascular reactivity such as flow-mediated dilation (FMD) responses and/or improved basal limb blood flow. However, the effect of BFR exercise on vascular reactivity is still controversial.

To date, three studies examined about acute effects of BFR walking [139] and resistance training with BFR [140, 141] on endothelial function assessed by FMD. Although exercise modality and target conduit artery vary, two of three studies showed impaired FMD with BFR exercise/training and one study indicated unaltered FMD with four-week training with BFR. Renzi et al. [139] demonstrated that FMD was impaired after BFR walking compared to walking without BFR. In their study, systemic cardiovascular responses, such as blood pressure, and total peripheral resistance significantly increased and stroke volume/pulse pressure (an index of systemic arterial compliance) significantly decreased during walking compared to those in the control condition while ABI showed a similar value.

In addition to this cross-sectional study, two studies on chronic BFR training showed different results in FMD responses. Credeur et al. [140] reported that four-weeks hand-grip training with BFR reduced FMD, whereas Hunt et al. [141] demonstrated that four-weeks hand-grip training with BFR did not alter FMD responses but induced transient adaptations to brachial artery structure, that is, increased resting and maximal vessel diameter. Although the mechanism underlying impaired FMD responses after acute BFR walking and chronic BFR training is still unclear, it is assumed that reduced FMD is a product of diminished endothelial function. Moreover, they speculated that oxidative stress induced by ischemic reperfusion injury might be the cause of the reduced FMD [139, 140]. As increased oxidative stress is associated with endothelial dysfunction [142, 143], followed by arteriosclerosis, augmented oxidative stress plays an important role in the pathogenesis and development of cardiovascular disease [144]. It has been reported that high-intensity resistance exercise, that is, >70% 1 RM, elicits oxidative stress markers [145–149], whereas these responses to lower-intensity exercise have been inconsistent [150, 151].

During BFR exercise, the effects of both exercise intensity and ischemic reperfusion need to be accounted for. As far as we know, only two studies have examined the effect of BFR exercise on oxidative stress markers. Takarada et al. [117] measured serum interleukin-6 (IL-6) and lipid peroxides (LP) before and after low-intensity resistance exercise (20% 1 RM) with moderate blood flow restriction and found no significant increases. Although exhaustive exercise in humans has been shown to cause a sustained elevation of muscular xanthine oxidase activity as well as increases in serum IL-6 and LP concentrations [152], it did not seem to produce an excess amount of oxygen-derived free radicals in Takarada et al.'s study [117]. It was also reported that protein carbonyls and blood glutathione, which are indicators of oxidative stress, did not increase during low-intensity (30% 1 RM) BFR exercise, but did increase in moderate-resistance exercise (up to 70% 1 RM) [153]. Since both of previous studies did not measure oxidative stress markers [139, 140], it is unknown whether impaired FMD responses are associated with elevated oxidative stress. However, the study of Credeur et al. [140] used higher-intensity resistance load (60% 1 RM) and applied the cuff for a longer occlusion time (20 min). Thus, there is a possibility that increased oxidative stress may have reduced the FMD in the case of Credeur's study [140] because it was reported that the region between 60 and 70% 1 RM may be a critical load marker [145–151], in terms of inducing oxidative stress during resistance training without BFR. Another explanation associated with impaired FMD responses after BFR walking is that elevated blood pressure during 20-min BFR walking. The blood pressure of subjects during 20-min walking with blood flow restriction in Renzi et al. [139] was >20% higher than controls, whereas Takano et al. [116] observed only slightly higher blood pressure during knee extension with BFR than that without BFR (127 versus 113 mmHg). Since acute elevations in arterial blood pressure are associated with the arterial structure and/or the arterial load-bearing properties of collagen and elastin [154], the rise in blood pressure during BFR walking might have caused the diminished FMD.

Collectively, although the influence of BFR walking and training with small muscle group on endothelial dysfunction assessed by FMD is still controversial, these contradictory results may be dependent on the variety of methodologies used, including exercise intensity and occlusion time during exercise. Moreover, it is very difficult to obtain consensus due to lack of evidence. However, it should be still noted that cuff release-induced hyperemic blood flow may be expected, suggesting that it stimulates shear stress, followed by nitric oxide increase, simultaneously, BFR exercise and training should be carefully prescribed because it is supposed that systemic arterial compliance increase during BFR walking [139].

### 5.3. Small Muscle Group Exercise and Training

As large muscle group dynamic exercise may alter not only local vasculature but also systemic cardiovascular responses to either acute or chronic exercise, small muscle group exercise may be useful as a physiological model to observe impact of exercise training on localized vasomotor control in skeletal muscles without marked changes in central hemodynamics during exercise. Thus, to date, several studies have used handgrip exercise training to elucidate vascular function. Accordingly, two studies of handgrip exercise training with BFR have been investigated, furthermore, studies of calf raise with own body weight and planter flexion exercise training with BFR were also carried out. Generally, handgrip exercise training increases forearm blood flow and/or improves FMD with one exceptional unchanged study. Enhanced brachial FMD responses as well as endothelium-dependent vasodilatation were observed after isometric handgrip training in old men [155], in elderly hypertensives [156, 157], and chronic heart failure subjects [158, 159]. Similarly, forearm blood flow was increased with regular handgrip exercise training in young men [160, 161] and in middle aged [162]. In contrast, improved blood flow was not observed in patients with chronic heart failure [162]. Moreover, McGowan et al. [163] reported that isometric handgrip training did not alter FMD with normal blood pressure. One interesting finding was that four-week handgrip exercise training improved peak vasodilator capacity induced by 10-minute ischemic stimulus without influencing endothelium vasodilator system [164]. These results suggested that evaluation for vascular function should be considered from many aspects, such as endothelium-dependent dilation, basal blood flow, and peak vasodilator capacity.

In addition to these previous studies of handgrip exercise training without BFR, two studies of handgrip exercise training with BFR have been elucidated, resulted in impaired [140] and unchanged [141] FMD responses. As above stated, exercise intensity, which was used in one previous study, was higher and longer, that is, 60% MVC dynamic handgrip exercise training for 20 min [140], compared to another previous study [141] with BFR (Tables 1 and 2). It is possible that these higher exercise intensity and longer duration may cause oxidative stress, resulted in diminished FMD responses. Interesting findings were that handgrip exercise training with BFR did not improve FMD responses but caused brachial artery structural modifications, that is, increased resting and maximal diameters [141]. Based on the principle of FMD calculation, changes in baseline diameter can influence the magnitude of FMD responses. Moreover, it should be taken a consideration about time course alterations in vessel structure and function. Animal studies showed that prolonged training induces structural changes, namely, “arterial remodeling” [165, 166], while short-term training improves vascular function via enhanced NO bioavailability [167–170]. Indeed, in human studies, brachial artery FMD increases after just one week of handgrip exercise training [171] and decreased after two weeks, whereas conduit artery vasodilator capacity showed a progressive increase for eight weeks during training intervention [172]. In one of the BFR study, FMD did not change after four weeks training, however, peak blood flow after ischemia and baseline diameter significantly increased [141]. It may be speculated that four-week BFR handgrip exercise training might cause improvement in functional changes within a few weeks, followed by structural changes after four weeks, resulting in without changes FMD responses normalized by increased basal vessel diameter.

### 5.4. Other Relevant Findings

It is well known that coagulation factors and fibrinolysis are closely associated with the risk of cardiovascular diseases such as ischemic heart disease, particularly acute coronary syndrome [173]. Several previous studies have demonstrated the influence of BFR training on coagulation factors and fibrinolysis. However, to date, no study has observed a significant increase in either acute [120, 174, 175] or chronic [128] BFR exercise. It was reported that thrombin generation is associated with exercise-induced metabolites [176, 177]. Indeed, BFR exercise produced greater metabolites [118]; hence, there is a possibility that increased metabolites might produce coagulation factors and fibrinolysis. In contrast, Hilberg et al. [178, 179] reported that thrombin antithrombin III complex (TAT) and *D*-dimer increased after 60–90 min prolonged exercise [179] but did not increase after high-intensity exercise [178], from which blood lactate concentrations were significantly increased, suggesting that the TAT and *D*-dimer may be dependent on exercise time. Thus, it is unlikely that accumulated metabolites during BFR would be related to coagulation factors and fibrinolysis. Another possible explanation is that expanded blood flow after cuff release would exclude thrombosis, so that the possibility of thrombosis during BFR could be ruled out [120]. More important findings were that the incidence of serious side effects related to thrombosis was lower in 7 cases (0.055%) based on the national survey for more than 12,000 people in Japan [180]. These results indicated relative safety and potent implications to various populations of BFR training.

It has been pointed out that endothelial dysfunction may be improved through angiogenesis induced by vascular endothelial growth factors [181]. One study revealed that acute BFR exercise significantly increased vascular endothelial growth factor (VEGF) compared to control exercise [116]. The underlying mechanism may be related to local muscle ischemia during BFR exercise. In fact, VEGF secretion and production are activated under hypoxia [182, 183] and/or under decreases in local muscle oxygen tension during exercise [184]. Increased VEGF may produce angiogenesis, followed by enhanced NO bioavailability [181], which might be a beneficial effect for endothelial function. However, since no studies have examined the effects of chronic BFR training on VEGF, it is uncertain whether chronic BFR training can increase VEFG, resulting in an improvement of endothelial function.

## 6. Summary and Future Perspectives

**Figure 1:**
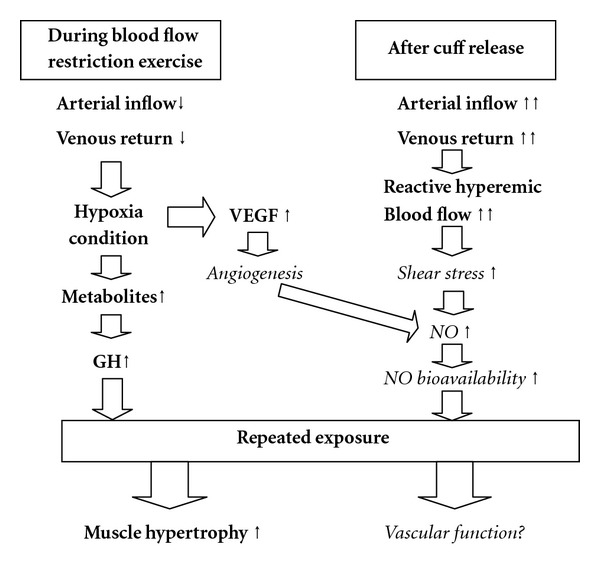
Hypothesised changes in vascular function in response to BFR exercise and training. BFR, blood flow restriction; GH, growth hormone; NO, nitric oxide; VEGF, vascular endothelial growth factor; Bold refers to known results from previous studies and Italic means unknown and inconsistent results form previous studies.

In this review, we focused on what is currently known (Tables 1 and 2) and hypothesised changes (Figure 1) of the influences of BFR exercise and training on vascular function. It is well established that aerobic exercise can improve vascular function but with insufficient muscle hypertrophy, while it is possible that high-intensity resistance training can produce muscle hypertrophy but with impaired vascular function. Higher load of resistance training should be considered carefully to apply aged people, disease patients, and rehabilitaining athletes. This paradoxical problem remains unsolved. BFR exercise and training might be a novel therapeutic modality because it combines lower exercise intensity than higher-intensity resistance training from the point view of during exercise with enhanced reactive hyperemic blood flow after cuff release. Although accumulating evidence has revealed that BFR training leads to muscle hypertrophy and strength increase as well as high-intensity resistance training, little attention has been given to the impact of BFR training on vascular function. At least the majority of the previous studies have demonstrated that BFR training did not impair arterial compliance. Conversely, the effect of BFR training and BFR walking on endothelial function assessed by FMD is not consistent. Available evidence suggests that acute BFR training did not increase oxidative stress markers or coagulation factors. Also, the effect of BFR training on vascular function may be influenced by various factors, such as, age, sex, exercise type, intensity, applied cuff pressure, and intervention period, not to mention the different evaluative methods for vascular function. However, the majority of BFR exercise and training use lower intensity, that is, 20–40% 1 RM, compared to high-intensity resistance training, for example, >80% 1 RM. High-intensity resistance training has a possibility to impair vascular function. Therefore, BFR exercise and training seems to become novel method because BFR exercise and training can obtain muscle hypertrophy as well as high-intensity resistance training even if BFR exercise and training does not affect vascular function. Future studies should be conducted with the aim of elucidating the mechanisms of the influence of BFR training on vascular function, with careful selection of the influencing parameters mentioned above and the potential therapeutic benefits for the elderly and for the rehabilitation of patients with cardiovascular disease and physical injury.

## Figures and Tables

**Table 1:** Acute effects of blood flow restricted exercise on vascular function and related biomarkers.

Author & year	Subjects	Age (yrs)	Applied cuff pressure (mmHg)	Intensity or velocity	Reps, sets, or time	Exercise modality	Occlusion time (min)	Cuff width (cm)	Outcome	Effect
Renzi et al. (2010) [139]	11 men 6 women	26	160	2 miles/h	10 min	walking	14	24	FMD	−
Fahs et al. (2011) [124]	11 men	28	200	20% 1 RM	30 reps + 15 reps ∗ 3	KE, KF	18	5	Arterial compliance Vascular conductance	+ ~
Fry et al. (2010) [120]	7 men	70	200	20% 1 RM	30 reps + 15 reps ∗ 3	KE	4-5	5	*D*-dimer	~
Madarame et al. (2010) [174]	10 men	25	150–160	30% 1 RM	30 reps + 15 reps ∗ 3	LP	?	5	PTF TAT *D*-dimer FDP	~ ~ ~ ~
Nakajima et al. (2007) [175]	6 men	32	160	30% 1 RM	30 reps + 15 reps ∗ 3	LP	?	5	*D*-dimer FDP Fibrinogen	~ ~ ~
Takano et al. (2005) [116]	11 men	34	160–180	20% 1 RM	30 reps ∗ 3 until exhaustion	KE	?	3.3	VEGF	+

RM: repetition maximum; KE: knee extension; KF: knee flexion; LP: leg press; FMD: flow mediate dilation; SV: stroke volume; PP: pulse pressure; PTF: prothrombin fragment 1 + 2; TAT: thrombin antithrombin III complex; FDP: fibrinogen degaradation product; VEGF: vascular endothelial growth factor. +: positive effect, −: negative effect, ~: no effect.

**Table 2:** Chronic effects of blood flow restricted training on vascular function and related biomarkers.

Author & year	Subjects	Age (yrs)	Applied cuff pressure (mmHg)	Intensity or time	Reps, sets, or time	Exercise modality	Intervention period	Occlusion time (min)	Cuff width (cm)	Outcome	Effect
Credeur et al. (2010) [140]	5 men 7 women	22	80	60% 1 RM	15 reps/min, 20 min	HG	3 days/wk 3 wk	20	?	FMD	−
Hunt et al. (2012) [141]	9 men	26	80	40% 1 RM	20 reps/min until exhaustion	HG	3 days/wk 4 wk	8.5	13	FMD Diameter	~ +
Clark et al. (2011) [128]	8 men 1 women	24	SBP ∗ 1.3	30% 1 RM	8–12 reps/set, Total 30–50 reps	KE	3 days/wk 3 wk	?	6	PWV, ABI *D*-dimer, PT Fibrinogen	~,~ ~,~ ~
Fahs et al. (2012) [129]	10 men	21	160–200	20% 1 RM	30 reps +15 reps ∗ 3 set	KE, KF	3 days/wk 6 wk	12	5	Arterial compliance Vascular conductance	~ ~
Kim et al. (2009) [127]	10 men	18–35	SBP ∗ 1.2 ∗ 1.2	20% 1 RM	10 reps ∗ 2	KE, KF, LP	3 days/wk 3 wk	<15	5	Arterial compliance	~
Ozaki et al. (2011) [134]	3 men 10 women	66	140–200	45% HRR	20 min	walking	4 days/wk 10 wk	20	5	Arterial compliance	+
Ozaki et al. (2012) [130]	10 men	22–32	160	30% 1 RM	30 reps + 15 reps ∗ 3 set	BeP	3 days/wk 6 wk	?	3	Arterial compliance	~
Patterson and Ferguson (2010) [119]	8 men 8 women	23 22	110	25%, 50% 1 RM	20 reps/min ∗ 3 sets	PF	3 days/wk 4 wk	5–8	?	Post-occlusive blood flow	+
Evans et al. (2010) [138]	9 men	20	150	Own BW	50 reps ∗ 4	CR	3 days/wk 4 wk	?	?	Microvascular filtration capacity	+

SBP: systolic blood pressure; HRR: heart rate reserve; BW: body weight; HG: hand grip; PF: planter flexion; CR: calf raise; BeP: Bench press; PWV: pulse wave velocity; ABI: ankle-brachial blood pressure index. PT: prothrombin time; +: positive effect; −: negative effect; ~: no effect.
